# Oral intake of heat-killed *Lactobacillus plantarum* L-137 decreases the incidence of upper respiratory tract infection in healthy subjects with high levels of psychological stress

**DOI:** 10.1017/jns.2013.35

**Published:** 2013-12-06

**Authors:** Yoshitaka Hirose, Yoshihiro Yamamoto, Yasunobu Yoshikai, Shinji Murosaki

**Affiliations:** 1 Research and Development Institute, House Wellness Foods Corp., Itami, Japan; 2 Research Center for Infectious Diseases, Medical Institute of Bioregulation, Kyushu University, Kyushu, Japan

**Keywords:** *Lactobacillus plantarum*, Immune function, Wisconsin Upper Respiratory Symptom Survey, Upper respiratory tract infections, Stress, Con A, concanavalin A, HK L-137, heat-killed *Lactobacillus plantarum* L-137, IFN, interferon, PBMC, peripheral blood mononuclear cells, Th, T helper, URTI, upper respiratory tract infection, WURSS, Wisconsin Upper Respiratory Symptom Survey, WURSS-21, short-form Wisconsin Upper Respiratory Symptom Survey, WURSS-44, long-form Wisconsin Upper Respiratory Symptom Survey

## Abstract

The immunomodulatory effects of live or non-viable lactic acid bacteria have been extensively investigated. We reported that oral intake of heat-killed *Lactobacillus plantarum* L-137 (HK L-137) augmented innate and acquired immunity in mice and human subjects. To examine the effects of HK L-137 intake on upper respiratory tract infection (URTI) symptoms and immune functions in human subjects, a randomised, double-blind, placebo-controlled, parallel study was conducted in subjects with high psychological stress levels. A total of seventy-eight healthy subjects (thirty-three men and forty-five women; mean age 50·6 years) with scores of >41 on eighteen-item subscales of psychological distress in the Brief Job Stress Questionnaire were randomly assigned to receive a tablet containing HK L-137 (10 mg) or a placebo tablet daily for 12 weeks. The URTI symptoms were rated once daily on the validated twenty-one-item Wisconsin Upper Respiratory Symptom Survey-21. Immune functions, such as concanavalin A-induced proliferation and percentages of interferon (IFN)-γ- and IL-4-producing CD4 T cells of peripheral blood mononuclear cells (PBMC), and serum IFN-β concentrations were measured every 4 weeks. URTI incidence was significantly lower in the HK L-137 group than in the control group. URTI incidence, duration and severity, and duration of medication showed significant negative correlations with duration of HK L-137 intake. The percentage change from baseline of concanavalin A-induced proliferation of PBMC was significantly greater in the HK L-137 group than in the control group. These findings suggest that daily HK L-137 intake can decrease URTI incidence in healthy subjects, possibly through augmentation of immune functions.

Lactobacilli, non-pathogenic Gram-positive inhabitants of the normal human intestine, are known as probiotics for their health-promoting effects, including enhancement of the immune system, protection against intestinal infection, reduction of serum cholesterol concentrations, and anticarcinogenic activities^(^^1^^)^. Although probiotics were originally defined as live microbial food components, non-viable microbes have also been shown to exhibit beneficial effects that are equivalent to, or even greater than, those of live microbes^(^^2^^,^^3^^)^. Recently, bacteria that promote health by driving mucosal immune mechanisms have been defined as immunobiotics^(^^4^^)^.

Heat-killed *Lactobacillus plantarum* L-137 (HK L-137), a strain isolated from fermented food, is a potent inducer of IL-12, which leads to a T helper (Th) 1-type immune response and subsequent anti-allergic or anti-tumour effects in mouse models^(^^5^^–^^7^^)^. We have illustrated previously that heat treatment of L-137 hardly changes bacterial cell size but enhances immunomodulatory activity and stability in simulated digestive juices^(^^8^^)^. Moreover, it was also reported that daily intake of HK L-137 enhances Th1-related immune functions, such as increased concanavalin A (Con A)-induced proliferation and percentages of interferon (IFN)-γ- and IL-4-producing CD4 T cells (Th1:Th2 ratio) of peripheral blood mononuclear cells (PBMC) in healthy subjects^(^^9^^)^. In a recent study, we demonstrated that oral administration of HK L-137 enhances protection against influenza virus infection by stimulation of type I IFN production in mice^(^^10^^)^. Furthermore, we observed increased production of type I IFN after intake of HK L-137 in human subjects^(^^11^^)^. These findings suggest that HK L-137 may boost host defence against common cold infections, including influenza A virus infection, in humans.

Physical and psychological stress is one of the factors that lowers immune functions, together with malnutrition, ageing, intense exercise and undesirable lifestyles^(^^12^^)^. It is now well established that the immune, endocrine and central nervous systems interact, and that psychological stress causes dysregulation of immune responses via effects on the hypothalamic–pituitary–adrenal axis and the autonomic nervous system^(^^13^^)^. Many studies have indicated that psychological stress score is strongly correlated with an increased risk of not only acute infectious respiratory illness, but also development of acute and chronic fatigue syndromes^(^^14^^–^^17^^)^. These observations suggest that individuals under high levels of psychological stress are susceptible to catching a cold.

The common cold is a clinical syndrome resulting from viral infection of the upper respiratory tract. Although upper respiratory tract infection (URTI) is extremely common, accounting for up to half of all acute illness episodes^(^^18^^)^, there are no perfect tools for assessing common cold infections, including URTI. The Wisconsin Upper Respiratory Symptom Survey (WURSS) was developed using individual interviews and focus groups among community-recruited individuals with Jackson-defined colds^(^^19^^)^. The semi-structured interviews included open-ended questions aimed at eliciting terminology and assessing health values related to experience of a cold-related illness. Of more than 150 terms used to define symptomatic or functional impairment, forty-four were chosen for inclusion in the long-form WURSS-44^(^^19^^)^ and twenty-one were selected for inclusion in a shorter form, the WURSS-21^(^^20^^)^. The WURSS-44 and WURSS-21 have been widely validated and confirmed to perform well as illness-specific quality-of-life evaluative outcome instruments. Therefore, these self-rating questionnaires may be good tools for assessing the symptoms of URTI.

The objective of the present study was to examine the effects of HK L-137 intake on the incidence and severity of URTI using the WURSS-21 in healthy subjects with high levels of psychological stress, in addition to its effects on immune functions.

## Experimental methods

### Subjects

**Fig. 1.**
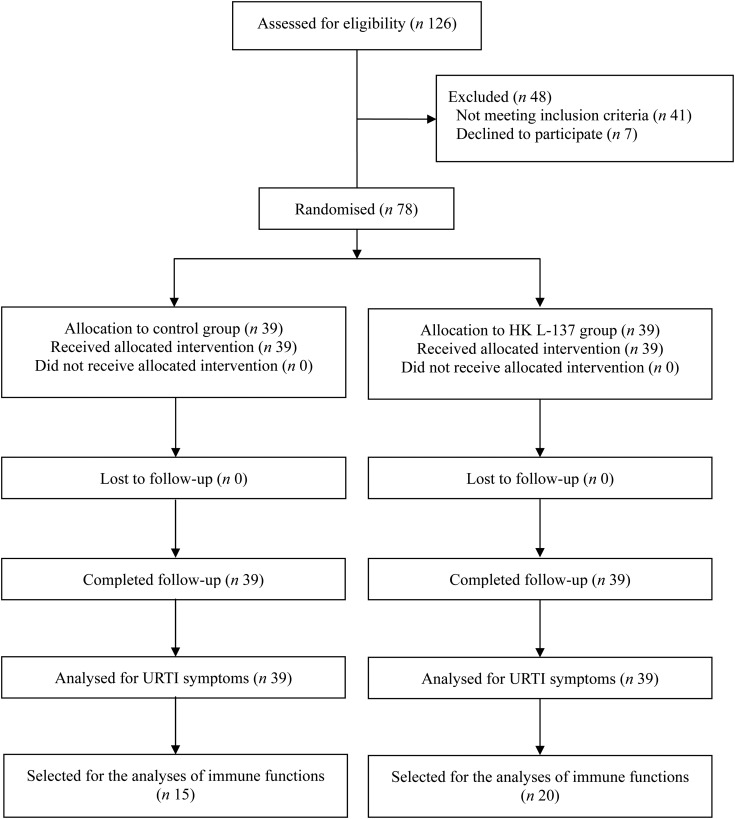
Subject flowchart summarised according to Consolidated Standards of Reporting Trials (CONSORT) that shows the number of subjects randomly assigned, lost to follow-up and analysed by treatment arm. HK L-137, heat-killed *Lactobacillus plantarum* L-137; URTI, upper respiratory tract infection.

Healthy subjects under stress aged 40–64 years were recruited from October 2011 to December 2011 and were assessed for eligibility to take part in the present study. Stress response in the subjects was examined by the Japan Brief Job Stress Questionnaire^(^^21^^)^. The questionnaire was developed by a stress assessment study group, ‘The Study Group for the Prevention of Work-Related Disease’, of the Japanese Ministry of Health, Labour and Welfare (1995–1998). It is used to assess total job stress with subscales of: (a) job stressor; (b) job stress response; (c) social support; and (d) degree of satisfaction. The job stress response includes six psychological (eighteen items on vigor, impatience, fatigue, anxiety and depression) stress response subscales, which were used in the present study. The subjects rated their response to each question from 1 (not at all) to 4 (yes, absolutely), and their psychological distress was estimated from the total score of the sum of the eighteen items (score range 18–72). It was reported that the total scores can be classified into five groups based on the evaluation of 11847 subjects: highest (51–72; 8·4 %); second highest (42–50; 19·4 %); moderate (33–41; 37·7 %); second lowest (24–32; 30·2 %); and lowest (18–23; 4·3 %)^(^^21^^)^. In accordance with this classification, subjects with total scores of >41 were defined as having high levels of psychological stress in the present study. The inclusion criterion was being a healthy subject with a score of >41 for the eighteen-item subscales of psychological distress in the Japan Brief Job Stress Questionnaire. Exclusion criteria included current history of illness including diabetes, liver disease, renal disease, cardiac disease, and inappropriate secretion of adrenal cortex hormone, or a previous history of these diseases, allergic disease or intolerance of milk, current treatment with a prescribed medicine, poor compliance with the clinical trial guidelines, and being an inappropriate subject as judged by the investigator. Among 126 healthy subjects recruited, seventy-eight subjects (thirty-three men and forty-five women; mean age 50·6 years) were eligible and randomly assigned (Fig. 1). Sample size was determined based on previous studies of the effects of probiotics on URTI^(^^22^^,^^23^^)^. A sample size estimation of sixty-four subjects was based on an expected rate of 2·0 (sd 1·0) URTI episodes during the winter months^(^^24^^)^, a target 25 % reduction in the number of episodes, statistical power of 80 %, and a type I error of 5 %. We initially recruited seventy-eight volunteers to account for an estimated 20 % drop-out rate over the study period. Eating habits were not considered for the criteria, but the subjects were instructed to continue their habitual diets during the study. Approval of the protocol was obtained from the Nihonbashi Cardiology Clinic Ethical Review Board (Tokyo, Japan), and the study was conducted in accordance with the Declaration of Helsinki of 1975 as revised in 2008 and with the ethical guidelines for epidemiological research proposed by the Ministry of Education, Culture, Sports, Science and Technology and the Ministry of Health, Labour and Welfare. The procedures were fully explained to the subjects, and written informed consent was obtained from each subject before the beginning of the study.

### Preparation of heat-killed *Lactobacillus plantarum* L-137

LP20 (House Wellness Foods Corporation), which contains 20 % HK L-137 and 80 % dextrin, was used in the present study. The preparation of HK L-137 for LP20 was carried out on the basis of a previously described method^(^^5^^)^.

### Experimental design

A total of seventy-eight healthy subjects were enrolled in a randomised, double-blind, placebo-controlled, parallel study. The subjects were randomly assigned to two groups using a sequential series of numbered sealed envelopes that each contained one food ingredient assigned in a computer-randomised manner. After the assignment, the subjects consumed either a tablet containing 50 mg of LP20 (one tablet daily) or a matching control tablet, in which LP20 was substituted with dextrin, for 12 weeks. Throughout the study, the subjects rated themselves once daily in a subject diary to assess their general health, duration of medication, and compliance with the dietary regimens, and by the self-rating questionnaire to examine URTI symptoms. Blood samples were collected every 4 weeks, and biomarkers for immune function were also measured every 4 weeks. Safety assessments, such as anthropometric measurements, biochemical examinations of blood, haematological assessments and urine tests, were performed at 0 and 12 weeks. Measurements of the biomarkers and blood and urine tests were completed by a clinical laboratory testing company, SRL. The study was conducted at Nihonbashi Cardiology Clinic (Tokyo, Japan) from December 2011 to March 2012.

### Upper respiratory tract infection symptoms

The incidence and severity of URTI symptoms were rated once daily on the WURSS-21 described in online Supplementary Appendix S1^(^^19^^,^^20^^)^, Japanese version. The first item, which reflected mental severity, was assessed separately. The subsequent nineteen items, which included ten items of cold symptoms and nine items of quality-of-life functional impairment, were used to evaluate the physical severity of URTI. The subjects answered the first item every day by marking a 0–7 Likert-type severity scale, where 0 = not sick, 1 = very mildly, 3 = mildly, 5 = moderately, and 7 = severely. The date of onset of cold illness was defined as the day of marking a number other than 0 after two previous markings of 0. If the subjects marked numbers other than 0 on the first or second day after the beginning of dietary intervention, the day was included as the date of onset. The scores for the incidence and duration of URTI were defined as the number of new onsets and the sum of days of marking numbers other than 0, respectively. The subjects who marked numbers other than 0 answered the following nineteen items by rating on a conventional Likert scale for cold symptoms, in which 0 = do not have this symptom, 1 = very mild, 3 = mild, 5 = moderate, and 7 = severe, and quality of life, in which 0 = not at all, 1 = very mildly, 3 = mildly, 5 = moderately, and 7 = severely. The scores of mental severity and physical severity were defined as the sums of the scales of the first item and of the following nineteen items, respectively.

### Medication

When the subjects used drugs during the study, they recorded the names of the drugs in the subject diary. If an anti-inflammatory drug was used on the day of marking a number other than zero in the first item, the drug use was defined as medication.

### Biomarkers for immune functions

PBMC were isolated by the Ficoll–Conray centrifugation technique (Ficoll–Conray, *d* = 1·077). PBMC (5 × 10^5^ cells/ml) were cultured with or without the optimal dose of Con A (5 µg/ml) for 72 h at 37°C, and pulse labelled with 37 kBq of [^3^H]thymidine during the last 8 h. DNA synthesis was assessed by measuring thymidine uptake. The percentages of IFN-γ- and IL-4-producing CD4^+^ T cells (Th1:Th2 ratio) were determined by single-cell measurements of intracellular cytokines using flow cytometry, as described^(^^25^^)^. Serum IFN-β concentrations were determined using an ELISA kit (PBL Biomedical Laboratories) according to the manufacturer's instructions.

### Statistical analysis

We compared the baseline values between the two groups using the unpaired *t* test for age, Con A-induced proliferation and Th1:Th2 ratio, and the Mann–Whitney *U* test for total psychological stress score. Because most serum IFN-β concentrations in subjects were below the detection limit, the difference in baseline concentrations in serum IFN-β between the two groups was also assessed by the Mann–Whitney *U* test. The scores for the incidence, duration, mental severity and physical severity of URTI, and duration of medication in each group were calculated every week and analysed by the Friedman test^(^^26^^,^^27^^)^. The correlations between the incidence, duration, mental severity and physical severity of URTI, and duration of medication and the duration of HK L-137 intake were analysed using Spearman's rank-correlation coefficient test. The percentage changes from baseline in the measurements of immune functions other than serum IFN-β concentration were analysed by two-way ANOVA, followed by comparisons between the two groups at each time point using the unpaired *t* test. The changes from baseline in the serum IFN-β concentrations between the two groups were assessed by the Mann–Whitney *U* test. All analyses were performed using PASW version 18.0J (SPSS Japan). A probability value of *P* < 0·05 was considered to indicate statistical significance.

## Results

### Baseline characteristics

All of the seventy-eight subjects completed the study and were included in the statistical analyses of the URTI symptoms. The baseline immune characteristics and psychological stress scores are shown in Table 1. None of the characteristics showed any differences between the two groups.

**Table 1.** Baseline psychological stress scores and immune characteristics of the subjects enrolled in the present study (Mean values and standard deviations)

	Control		HK L-137		All subjects	
	Mean	sd	Mean	sd	Mean	sd
Subjects (*n*)	39		39		78	
Sex (*n*)						
Male	14		19		33	
Female	25		20		45	
Age (years)	51·5	7·2	49·6	6·8	50·6	7·0
Immune function						
Unstimulated proliferation (×10^3^ cpm)	0·23	0·12	0·22	0·09	0·23	0·11
Con A-induced proliferation (×10^3^ cpm)	29·3	6·9	28·4	9·0	28·8	8·0
IFN-γ-producing cells (Th1 cells) (% CD4^+^ cells)	22·6	8·1	23·4	7·0	23·0	7·5
IL-4-producing cells (Th2 cells) (% CD4^+^ cells)	2·9	1·2	2·6	1·0	2·7	1·1
Th1:Th2 ratio	9·2	5·2	10·4	5·2	9·8	5·2
Serum IFN-β (IU/ml)*	11·3	35·1	17·4	49·3	14·3	42·6
Total psychological stress scores*	59·6	4·4	58·5	4·0	59·0	4·2

HK L-137, heat-killed *Lactobacillus plantarum* L-137; Con A, concanavalin A; cpm, counts per min; IFN, interferon; Th1, type 1 T helper; Th2, type 2 T helper.

* Compared between the two groups by the Mann–Whitney *U* test.

### Effects of dietary intervention on upper respiratory tract infection symptoms

During the study, twenty-four subjects in the control group contracted URTI with a total of forty-seven times, and nineteen subjects in the HK L-137 group contracted URTI with a total of thirty-five times. The incidence of URTI during the study was significantly lower in the HK L-137 group than in the control group (*P* = 0·011), while no differences in the other endpoints were observed between the two groups (Table 2). The incidence (*P* = 0·029), duration (*P* = 0·005), mental severity (*P* = 0·005) and physical severity (*P* = 0·007) of URTI, and duration of medication (*P* = 0·008) showed significant negative correlations with duration of HK L-137 intake (Table 2).

**Table 2.** Scores for incidence, duration, mental severity and physical severity of upper respiratory tract infection (URTI), and duration of medication in subjects with daily intake of a tablet containing 10 mg of heat-killed *Lactobacillus plantaru m* L-137 (HK L-137) or a control tablet*

		Intervention period												Friedman test: *P*†		Spearman's rank-correlation coefficient‡	
End point		1 week	2 weeks	3 weeks	4 weeks	5 weeks	6 weeks	7 weeks	8 weeks	9 weeks	10 weeks	11 weeks	12 weeks	Intervention	Time point	Correlation coefficient	*P*
Incidence (number of cases)	Control	7	6	5	3	3	4	3	4	2	3	5	2	0·011	0·075	−0·619	0·032
	HK L-137	6	5	4	6	0	2	3	3	0	1	3	2			−0·627	0·029
Duration (d)	Control	20	16	8	24	9	10	6	14	10	7	23	12	1·000	0·172	−0·165	0·609
	HK L-137	19	23	24	31	15	8	12	15	0	4	11	9			−0·753	0·005
Mental severity	Control	37	28	10	30	9	22	11	26	22	11	40	17	0·564	0·445	−0·098	0·761
	HK L-137	31	73	58	49	36	13	22	38	0	14	13	10			−0·776	0·005
Physical severity	Control	280	271	56	205	24	296	54	171	115	65	249	137	0·564	0·443	−0·224	0·484
	HK L-137	209	953	883	492	202	137	226	273	0	169	104	42			−0·734	0·007
Duration of medication (d)	Control	10	12	2	3	1	6	1	4	6	3	3	7	0·132	0·548	−0·117	0·718
	HK L-137	7	6	8	7	6	0	0	2	0	3	1	0			−0·722	0·008

* Control group, *n* 39; HK L-137 group, *n* 39.

† Significant differences estimated between the two groups at the time points.

‡ Correlations between the symptoms of URTI, and duration of medication and duration of control tablet or HK L-137 intake.

### Effects of dietary intervention on immune functions

The subjects who contracted URTI during the intervention were excluded from the analyses of immune functions, because the symptoms of URTI can considerably affect immunity. As a result, a total of thirty-five subjects were included in the statistical analyses of immune functions (Fig. 1). The percentage change in Con A-induced proliferation was significantly higher in the HK L-137 group than in the control group during the study (*P* = 0·048), whereas no difference was observed in the percentage change of unstimulated proliferation between the two groups (Table 3). The percentage changes in the Th1:Th2 ratios and the amounts of change in the serum IFN-β concentrations showed no differences between the two groups (Table 3).

**Table 3.** Percentage changes in immune characteristics from baseline in subjects with daily intake of a tablet containing 10 mg of heat-killed *Lactobacillus plantarum* L-137 (HK L-137) or a control tablet without upper respiratory tract infection (URTI) symptoms throughout the intervention period* (Mean values and standard deviations)

		Change from baseline (%)						ANOVA: *P*†		
		4 weeks		8 weeks		12 weeks		Intervention	Time point	Interaction‡
Unstimulated proliferation ( × 10^3^ cpm)	Control	22·5	73·7	8·1	37·9	–0·5	38·2	0·425	0·016	0·566
	HK L-137	29·9	90·1	–5·3	34·9	–21·4	37·1			
Con A-induced proliferation ( × 10^3^ cpm)	Control	−22·4	18·1	–13·1	22·0	−7·8	28·5	0·048	0·535	0·679
	HK L-137	−3·8	28·9	–3·9	37·4	–0·9	32·4			
IFN-γ-producing cells (Th1 cells) (%CD4^+^ cells)	Control	9·1	18·1	1·6	12·6	8·5	17·6	0·455	0·360	0·883
	HK L-137	5·8	20·1	1·3	14·0	4·1	21·3			
IL-4-producing cells (Th2 cells) (%CD4^+^ cells)	Control	18·2	41·6	13·5	44·3	−4·1	39·9	0·870	0·010	0·879
	HK L-137	20·1	45·0	14·7	40·9	−10·9	21·5			
Th1:Th2 ratio	Control	6·6	48·0	4·2	49·1	35·3	70·7	0·449	0·048	0·938
	HK L-137	1·3	51·6	–0·4	39·6	22·9	38·1			
Serum IFN-β	Control	0·2	6·8	−0·5	1·3	0·9	4·5	–	–	–
	HK L-137	−5·4	14·6	−6·7	31·3	−6·1	29·5			

cpm, Counts per min; Con A, concanavalin A; IFN, interferon; Th1, type 1 T helper; Th2, type 2 T helper.

* Control group: *n* 15; HK-LP group: *n* 20.

† Significant differences between the two groups at the time points were evaluated by two-way ANOVA. Serum IFN-β concentrations were compared between the two groups at each time point by the Mann–Whitney *U* test.

‡ Interaction between intervention and time point.

### Safety assessment

A total of 151 adverse events related to subjective or objective symptoms were recorded during the study. Of the seventy-three adverse events in the control group, forty-seven were symptoms of a common cold, fourteen were digestive symptoms, eight were pain, two were fatigue, one was furuncle, and one was foot corn. Of the fifty-eight adverse events in the HK L-137 group, thirty-five were symptoms of a common cold, eight were fatigue, seven were digestive symptoms, six were pain, one was furuncle, and one was chalazion. All adverse events were mild, and were judged to be unrelated to the dietary intervention. Among the safety assessments performed, albumin and diastolic blood pressure were significantly higher and lactate dehydrogenase was significantly lower in the HK L-137 group than in the control group, but the changes were within the ranges of the corresponding reference values.

## Discussion

In the present study, the healthy subjects with high levels of psychological stress who took HK L-137 daily had a significantly lower incidence of URTI throughout the study. In addition, the incidence, duration and severity of URTI, and duration of medication showed significant negative correlations with duration of HK L-137 intake. Among the immune functions, the percentage change from baseline in Con A-induced proliferation was significantly greater in the HK L-137 group than in the control group. These findings suggest the possibility that daily intake of HK L-137 decreases the incidence of URTI through augmentation of immune functions in healthy subjects.

Several lines of evidence have shown that dietary consumption of live or heat-killed lactic acid bacteria can alleviate the symptoms of URTI in human subjects. These studies were conducted in children^(^^28^^)^, elderly individuals^(^^29^^–^^33^^)^ and athletes^(^^22^^,^^23^^)^ whose immune functions were not fully developed, decreased with age and decreased with intense exercise, respectively. In the present study, we recruited subjects under high levels of psychological stress, who have also been reported to have weak immune functions and to catch a cold easily^(^^14^^–^^16^^)^. URTI is generally evaluated after diagnosis by a doctor or a self-rating questionnaire in clinical studies. However, there remain problems associated with diagnosing the symptoms of URTI in all subjects every day by the same doctor or validating the self-rating questionnaire for URTI used in each study. The WURSS-21 has been widely validated and confirmed to perform well as an illness-specific quality-of-life evaluative outcome instrument^(^^20^^)^, and we therefore used it to assess the symptoms of URTI in the present study.

We previously reported that healthy subjects with daily intake of HK L-137 showed sustained increases in Con A-induced proliferation of PBMC, Th1:Th2 ratio of CD4^+^ T cells^(^^9^^)^ and serum IFN-β concentration^(^^11^^)^ from 4 weeks, and that the health-related quality of life also improved from 8 weeks after the start of dietary intervention^(^^9^^)^. In the present study, the incidence of URTI decreased over time in the control group as well as in the HK L-137 group. It is possible that the epidemic period of seasonal common cold corresponded to the early part of the dietary intervention, December 2011 to January 2012, so the incidence of URTI decreased in all subjects. However, the incidence of URTI during the study was significantly lower in the HK L-137 group than in the control group. The other endpoints such as duration, severity, and duration of medication showed no differences between the two groups. These results may be influenced by differences in the timing or frequency of usage of anti-inflammatory drugs between the two groups. Nevertheless, all endpoints showed significant negative correlations with duration of HK L-137 intake, suggesting that continued intake of HK L-137 appears to be effective for not only the incidence but also the symptoms of URTI.

Immune functions were evaluated in a total of thirty-five subjects who did not contract URTI during the study and whose immune functions were unlikely to be affected by URTI. Among the immune functions, the percentage change in Con A-induced proliferation was significantly higher in the HK L-137 group than in the control group during the study, consistent with our previous findings. However, the percentage changes in the Th1:Th2 ratios and the amounts of change in the serum IFN-β concentrations showed no differences between the two groups. It is well established that psychological stress causes dysregulation of the immune response via effects on the hypothalamic–pituitary–adrenal axis and the sympathetic nervous system^(^^13^^)^. The earliest effect has been identified on the central nervous system-mediated immunoregulatory function involved in the brain's ability to suppress the transcription of both pro-inflammatory genes (such as *IL1B*, *IL6* and *TNF*) and antiviral genes (such as *IFNA* and *IFNB*) by stimulating glucocorticoid release from the hypothalamic–pituitary–adrenal axis^(^^34^^–^^36^^)^. Conversely, in the sympathetic nervous system, noradrenaline has been shown to modulate leucocyte gene expression via stimulation of β-adrenergic receptors, which are associated with the signalling cascade involving Gα_S_, adenylyl cyclase, cyclic AMP and proteinase A^(^^37^^)^. β-Adrenergic signalling has been indicated to modulate adaptive immune responses by stimulating the transcription of Th2-type cytokine genes (such as *IL4* and *IL5*) and suppressing the expression of Th1-type cytokine genes (such as *IFNG* and *IL12B*)^(^^38^^–^^40^^)^. Recent studies have discovered a similar sympathetic nervous system-mediated suppression of innate immune programmes, involving the suppression of type I IFN-mediated antiviral responses^(^^41^^)^ and up-regulated transcription of pro-inflammatory cytokine genes^(^^42^^–^^44^^)^. Therefore, it is possible that the induction of antiviral (such as IFN-α and IFN-β) or Th1-type (such as IFN-γ and IL-12) cytokines is strongly suppressed in subjects under high levels of psychological stress, resulting in reduced responses to HK L-137 intake for inducing IFN-β and IFN-γ in the subjects in the present study. Crucial roles for not only CD8^+^ T cells but also CD4^+^ T cells in viral infection have been reported^(^^45^^,^^46^^)^. Con A-induced T cell proliferation decreases greatly after endurance race events^(^^47^^)^, and endurance athletes are at increased risk for URTI after marathon-type race events for up to 2 weeks^(^^48^^)^. Other studies have indicated that phytohaemagglutinin-induced T cell proliferation is a good tool for determining the risk of further infection in adult transplant recipients^(^^49^^–^^51^^)^. The proliferative response of T cells in the HK L-137 group was enhanced in the present study, which can contribute to reductions in the incidence and severity of URTI.

In the measurements of unstimulated proliferation, percentage of IL-4-producing cells, or Th1:Th2 ratio, a highly significant time point effect was observed (Table 3). Because the methods for measurement of these biomarkers are based on a highly complicated process including cell culture, measurement noise must be considered. Intra-assay variation is thought to be lower in measurements at the same time point than in those at different time points. In addition, measurement reliability can be enhanced by measuring a control specimen. Therefore, we consider that comparison between groups at the same time point is reliable but that comparison over time in these cases is not.

In conclusion, we have demonstrated that daily intake of HK L-137 decreases the incidence of URTI in healthy subjects in parallel with augmentation of immunity. The present results suggest that intake of HK L-137 may be useful for the prevention and treatment of URTI in healthy individuals.

## Supplementary Material

Supplementary Material Supplementary information supplied by authors.

## Supplementary material

To view supplementary material for this article, please visit http://dx.doi.org/10.1017/jns.2013.35

## References

[1] Nomoto K (2008) Prevention of postoperative microbial infection by synbiotics. Indian J Exp Biol 46, 557–561 18814482

[2] Isolauri E, Kirjavainen PV & Salminen S (2002) Probiotics: a role in the treatment of intestinal infection and inflammation? Gut 50, Suppl. 3, III54–III59 11953334 10.1136/gut.50.suppl_3.iii54 PMC1867676

[3] Yasui N, Watanabe M, Iwao Y, (1999) Laparoscopically assisted bowel resection for primary mucosa-associated lymphoid tissue lymphoma of the cecum. Surg Laparosc Endosc Percutan Tech 9, 156–159 11757546

[4] Clancy R (2003) Immunobiotics and the probiotic evolution. FEMS Immunol Med Microbiol 38, 9–12 12900049 10.1016/S0928-8244(03)00147-0

[5] Murosaki S, Yamamoto Y, Ito K, (1998) Heat-killed *Lactobacillus plantarum* L-137 suppresses naturally fed antigen-specific IgE production by stimulation of IL-12 production in mice. J Allergy Clin Immunol 102, 57–64 9679848 10.1016/s0091-6749(98)70055-7

[6] Murosaki S, Muroyama K, Yamamoto Y, (2000) Antitumor effect of heat-killed *Lactobacillus plantarum* L-137 through restoration of impaired interleukin-12 production in tumor-bearing mice. Cancer Immunol Immunother 49, 157–164 10881695 10.1007/s002620050615 PMC11036971

[7] Hirose Y, Murosaki S, Fujiki T, (2010) Lipoteichoic acids on *Lactobacillus plantarum* cell surfaces correlate with induction of interleukin-12p40 production. Microbiol Immunol 54, 143–151 20236424 10.1111/j.1348-0421.2009.00189.x

[8] Fujiki T, Hirose Y, Yamamoto Y, (2012) Enhanced immunomodulatory activity and stability in simulated digestive juices of *Lactobacillus plantarum* L-137 by heat treatment. Biosci Biotechnol Biochem 76, 918–922 22738959 10.1271/bbb.110919

[9] Hirose Y, Murosaki S, Yamamoto Y, (2006) Daily intake of heat-killed *Lactobacillus plantarum* L-137 augments acquired immunity in healthy adults. J Nutr 136, 3069–3073 17116721 10.1093/jn/136.12.3069

[10] Maeda N, Nakamura R, Hirose Y, (2009) Oral administration of heat-killed *Lactobacillus plantarum* L-137 enhances protection against influenza virus infection by stimulation of type I interferon production in mice. Int Immunopharmacol 9, 1122–1125 19410659 10.1016/j.intimp.2009.04.015

[11] Arimori Y, Nakamura R, Hirose Y, (2012) Daily intake of heat-killed *Lactobacillus plantarum* L-137 enhances type I interferon production in healthy humans and pigs. Immunopharmacol Immunotoxicol 34, 937–943 22468623 10.3109/08923973.2012.672425

[12] Kaminogawa S & Nanno M (2004) Modulation of immune functions by foods. Evid Based Complement Alternat Med 1, 241–250 15841257 10.1093/ecam/neh042 PMC538513

[13] Irwin MR & Cole SW (2011) Reciprocal regulation of the neural and innate immune systems. Nat Rev Immunol 11, 625–632 21818124 10.1038/nri3042 PMC3597082

[14] Cohen S, Tyrrell DA & Smith AP (1991) Psychological stress and susceptibility to the common cold. N Engl J Med 325, 606–612 1713648 10.1056/NEJM199108293250903

[15] Cohen S, Tyrrell DA & Smith AP (1993) Negative life events, perceived stress, negative affect, and susceptibility to the common cold. J Pers Soc Psychol 64, 131–140 8421249 10.1037//0022-3514.64.1.131

[16] Cohen S, Frank E, Doyle WJ, (1998) Types of stressors that increase susceptibility to the common cold in healthy adults. Health Psychol 17, 214–223 9619470 10.1037//0278-6133.17.3.214

[17] Kerr JR & Mattey DL (2008) Preexisting psychological stress predicts acute and chronic fatigue and arthritis following symptomatic parvovirus B19 infection. Clin Infect Dis 46, e83–e87 18419428 10.1086/533471

[18] Douglas RM (1999) Respiratory tract infections as a public health challenge. Clin Infect Dis 28, 192–194 10064224 10.1086/515112

[19] Barrett B, Locken K, Maberry R, (2002) The Wisconsin Upper Respiratory Symptom Survey: development of an instrument to measure the common cold. J Fam Pract 51, 265–273 11978238

[20] Barrett B, Brown RL, Mundt MP, (2009) Validation of a short form Wisconsin Upper Respiratory Symptom Survey (WURSS-21). Health Qual Life Outcomes 7, 76. 10.1186/1477-7525-7-76 PMC2748069 19674476

[21] Shimomitsu T (2000) The final development of the Brief Job Stress Questionnaire mainly used for assessment of the individuals In Ministry of Labour Sponsored Grant for the Prevention of Work-Related Illness: The 1999 Report, pp. 126–164 [ M Kato , editor]. Tokyo: Tokyo Medical College

[22] Gleeson M, Bishop NC, Oliveira M, (2011) Daily probiotic's (*Lactobacillus casei* Shirota) reduction of infection incidence in athletes. Int J Sport Nutr Exerc Metab 21, 55–64 21411836 10.1123/ijsnem.21.1.55

[23] West NP, Pyne DB, Cripps AW, (2011) *Lactobacillus fermentum* (PCC^®^) supplementation and gastrointestinal and respiratory-tract illness symptoms: a randomised control trial in athletes. Nutr J 10, 30. 10.1186/1475-2891-10-30 PMC3083335 21477383

[24] Neville V, Gleeson M & Folland JP (2008) Salivary IgA as a risk factor for upper respiratory infections in elite professional athletes. Med Sci Sports Exerc 40, 1228–1236 18580401 10.1249/MSS.0b013e31816be9c3

[25] Openshaw P, Murphy EE, Hosken NA, (1995) Heterogeneity of intracellular cytokine synthesis at the single-cell level in polarized T helper 1 and T helper 2 populations. J Exp Med 182, 1357–1367 7595206 10.1084/jem.182.5.1357 PMC2192216

[26] Friedman M (1937) The use of ranks to avoid the assumption of normality implicit in the analysis of variance. J Am Stat Assoc 32, 675–701

[27] Conover WJ & Iman RL (1981) Rank transformation as a bridge between parametric and non-parametric statistics. Am Statistician 35, 124–129

[28] Hatakka K, Savilahti E, Ponka A, (2001) Effect of long term consumption of probiotic milk on infections in children attending day care centres: double blind, randomised trial. BMJ 322, 1327. 10.1136/bmj.322.7298.1327 PMC32161 11387176

[29] Fukushima Y, Miyaguchi S, Yamano T, (2007) Improvement of nutritional status and incidence of infection in hospitalised, enterally fed elderly by feeding of fermented milk containing probiotic *Lactobacillus johnsonii* La1 (NCC533). Br J Nutr 98, 969–977 17617944 10.1017/S0007114507764723

[30] Guillemard E, Tondu F, Lacoin F (2010) Consumption of a fermented dairy product containing the probiotic *Lactobacillus casei* DN-114001 reduces the duration of respiratory infections in the elderly in a randomised controlled trial. Br J Nutr 103, 58–68 19747410 10.1017/S0007114509991395

[31] Makino S, Ikegami S, Kume A, (2010) Reducing the risk of infection in the elderly by dietary intake of yoghurt fermented with *Lactobacillus delbrueckii* ssp. bulgaricus OLL1073R-1. Br J Nutr 104, 998–1006 20487575 10.1017/S000711451000173X

[32] Van Puyenbroeck K, Hens N, Coenen S, (2012) Efficacy of daily intake of *Lactobacillus casei* Shirota on respiratory symptoms and influenza vaccination immune response: a randomized, double-blind, placebo-controlled trial in healthy elderly nursing home residents. Am J Clin Nutr 95, 1165–1171 22440853 10.3945/ajcn.111.026831

[33] Shinkai S, Toba M, Saito T, (2013) Immunoprotective effects of oral intake of heat-killed *Lactobacillus pentosus* strain b240 in elderly adults: a randomised, double-blind, placebo-controlled trial. Br J Nutr 109, 1856–1865 22947249 10.1017/S0007114512003753

[34] Besedovsky H, del Rey A, Sorkin E (1986) Immunoregulatory feedback between interleukin-1 and glucocorticoid hormones. Science 233, 652–654 3014662 10.1126/science.3014662

[35] Sapolsky R, Rivier C, Yamamoto G, (1987) Interleukin-1 stimulates the secretion of hypothalamic corticotropin-releasing factor. Science 238, 522–524 2821621 10.1126/science.2821621

[36] Berkenbosch F, van Oers J, del Rey A, (1987) Corticotropin-releasing factor-producing neurons in the rat activated by interleukin-1. Science 238, 524–526 2443979 10.1126/science.2443979

[37] Nance DM & Sanders VM (2007) Autonomic innervation and regulation of the immune system (1987–2007). Brain Behav Immun 21, 736–745 17467231 10.1016/j.bbi.2007.03.008 PMC1986730

[38] Lee HJ, Takemoto N, Kurata H, (2000) GATA-3 induces T helper cell type 2 (Th2) cytokine expression and chromatin remodeling in committed Th1 cells. J Exp Med 192, 105–115 10880531 10.1084/jem.192.1.105 PMC1887713

[39] Panina-Bordignon P, Mazzeo D, Lucia PD, (1997) Beta2-agonists prevent Th1 development by selective inhibition of interleukin 12. J Clin Invest 100, 1513–1519 9294119 10.1172/JCI119674 PMC508332

[40] Cole SW, Korin YD, Fahey JL (1998) Norepinephrine accelerates HIV replication via protein kinase A-dependent effects on cytokine production. J Immunol 161, 610–616 9670934

[41] Collado-Hidalgo A, Sung C & Cole S (2006) Adrenergic inhibition of innate anti-viral response: PKA blockade of type I interferon gene transcription mediates catecholamine support for HIV-1 replication. Brain Behav Immun 20, 552–563 16504464 10.1016/j.bbi.2006.01.005

[42] Cole SW, Arevalo JM, Takahashi R, (2010) Computational identification of gene–social environment interaction at the human IL6 locus. Proc Natl Acad Sci U S A 107, 5681–5686 20176930 10.1073/pnas.0911515107 PMC2851818

[43] Grebe KM, Takeda K, Hickman HD, (2009) Cutting edge: sympathetic nervous system increases proinflammatory cytokines and exacerbates influenza A virus pathogenesis. J Immunol 184, 540–544 20018617 10.4049/jimmunol.0903395 PMC2941093

[44] Cole SW, Hawkley LC, Arevalo JM, (2007) Social regulation of gene expression in human leukocytes. Genome Biol 8, R189. 10.1186/gb-2007-8-9-r189 PMC2375027 17854483

[45] Matloubian M, Concepcion RJ & Ahmed R (1994) CD4^+^ T cells are required to sustain CD8^+^ cytotoxic T-cell responses during chronic viral infection. J Virol 68, 8056–8063 7966595 10.1128/jvi.68.12.8056-8063.1994 PMC237269

[46] Shedlock DJ & Shen H (2003) Requirement for CD4 T cell help in generating functional CD8 T cell memory. Science 300, 337–339 12690201 10.1126/science.1082305

[47] Nieman DC (1997) Immune response to heavy exertion. J Appl Physiol 82, 1385–1394 9134882 10.1152/jappl.1997.82.5.1385

[48] Nieman DC (1997) Risk of upper respiratory tract infection in athletes: an epidemiologic and immunologic perspective. J Athl Train 32, 344–349 16558471 PMC1320353

[49] Blazik M, Hutchinson P, Jose MD, (2005) Leukocyte phenotype and function predicts infection risk in renal transplant recipients. Nephrol Dial Transplant 20, 2226–2230 16030032 10.1093/ndt/gfi007

[50] Rodrigo E, Lopez-Hoyos M, Corral M, (2012) ImmuKnow as a diagnostic tool for predicting infection and acute rejection in adult liver transplant recipients: a systematic review and meta-analysis. Liver Transpl 18, 1245–1253 22740321 10.1002/lt.23497

[51] Moon HH, Kim TS, Roh YN, (2012) Can immune function assay predict infection or recovery? Transplant Proc 44, 1048–1051 22564622 10.1016/j.transproceed.2012.04.001

