# Supplementary material for: Oral intake of heat-killed Lactobacillus plantarum L-137 decreases the incidence of upper respiratory tract infection in healthy subjects with high levels of psychological stress
Source: J Nutr Sci. 2013 Dec 6;2:e39. doi: 10.1017/jns.2013.35 (PMC4153334; doi:10.1017/jns.2013.35)
Supplement: Supplementary Material — Supplementary information supplied by authors. [file S2048679013000359sup001.doc]

**HIROSE: SupplementaryAppendix S1**

***Wisconsin Upper Respiratory Symptom Survey – 21 ---*** Daily Symptom Report

| *Day:* | *Date:* | *Time:* | *ID*: |
| --- | --- | --- | --- |

**Please fill in one circle for each of the following items:**

|  | **Not sick** | Very mildly |  | Mildly |  | Moderately | | |  | Severely | |
| --- | --- | --- | --- | --- | --- | --- | --- | --- | --- | --- | --- |
|  | **0** | **1** | **2** | **3** | **4** | | **5** | **6** | | | **7** |
| How sick do you feel **today**? | O | O | O | O | O | | O | O | | | O |

Please rate the **average severity of your cold symptoms over the last 24 hours** for each symptom:

|  | **Do not have  this symptom** | | Very mild |  | Mild |  | Moderate | | |  | Severe | |
| --- | --- | --- | --- | --- | --- | --- | --- | --- | --- | --- | --- | --- |
|  | | **0** | **1** | **2** | **3** | **4** | | **5** | **6** | | | **7** |
| Runny nose | | O | O | O | O | O | | O | O | | | O |
| Plugged nose | | O | O | O | O | O | | O | O | | | O |
| Sneezing | | O | O | O | O | O | | O | O | | | O |
| Sore throat | | O | O | O | O | O | | O | O | | | O |
| Scratchy throat | | O | O | O | O | O | | O | O | | | O |
| Cough | | O | O | O | O | O | | O | O | | | O |
| Hoarseness | | O | O | O | O | O | | O | O | | | O |
| Head congestion | | O | O | O | O | O | | O | O | | | O |
| Chest congestion | | O | O | O | O | O | | O | O | | | O |
| Feeling tired | | O | O | O | O | O | | O | O | | | O |

**Over the last 24 hours,** how much has your cold interfered with your ability to:

|  | **Not  at all** | Very mildly |  | Mildly |  | Moderately | | |  | Severely | |
| --- | --- | --- | --- | --- | --- | --- | --- | --- | --- | --- | --- |
|  | **0** | **1** | **2** | **3** | **4** | | **5** | **6** | | | **7** |
| Think clearly | O | O | O | O | O | | O | O | | | O |
| Sleep well | O | O | O | O | O | | O | O | | | O |
| Breathe easily | O | O | O | O | O | | O | O | | | O |
| Walk, climb stairs, exercise | O | O | O | O | O | | O | O | | | O |
| Accomplish daily activities | O | O | O | O | O | | O | O | | | O |
| Work outside the home | O | O | O | O | O | | O | O | | | O |
| Work inside the home | O | O | O | O | O | | O | O | | | O |
| Interact with others | O | O | O | O | O | | O | O | | | O |
| Live your personal life | O | O | O | O | O | | O | O | | | O |

**Compared to yesterday**, I feel that my cold is…

| Very much better | Somewhat better | A little  better | The same | A little  worse | Somewhat worse | Very much worse |
| --- | --- | --- | --- | --- | --- | --- |
| O | O | O | O | O | O | O |

WURSS -21© (Wisconsin Upper Respiratory Symptom Survey) 2004

Created by Bruce Barrett MD PhD et al., UW Department of Family Medicine, 777 S. Mills St. Madison, WI 53715, USA
